# Azido­(benzonitrile-κ*N*)[hydrido­tris(pyrazol-1-yl-κ*N* ^2^)borato](triphenyl­phosphine-κ*P*)ruthenium(II)

**DOI:** 10.1107/S1600536810021513

**Published:** 2010-06-30

**Authors:** Chiung-Cheng Huang, Han-Gung Chen, Yih Hsing Lo, Wen-Rong Lai, Chia-Her Lin

**Affiliations:** a Department of Chemical Engineering, Tatung University, Taipei 104, Taiwan; b Department of Natural Science, Taipei Municipal University of Education, Taipei 10048, Taiwan; c Department of Chemistry, Chung-Yuan Christian University, Chung-Li 320, Taiwan

## Abstract

Facile ligand substitution is observed when the ruthenium–azide complex, [RuN_3_(Tp)(PPh_3_)_2_] [Tp,HB(pz)_3_, pz = pyrazol­yl, PPh_3_ = triphenyl­phosphine] is treated with benzo­nitrile, yielding the title compound, [Ru(C_9_H_10_BN_6_)(N_3_)(C_7_H_5_N)(C_18_H_15_P)]. The asymmetric unit contains two crystallographically independent mol­ecules. In each one, the Ru^II^ atom is six-coordinated in a distorted octa­hedral geometry by five N atoms from an htpb ligand, an azide ligand and a benzonitrile ligand and one P atom from a triphenyl­phosphine (tpp) ligand. The azide group is almost linear and is coordinated to Ru with an average Ru—N—N angle of 124.9 (3)°.

## Related literature

For general background to azide and triazole compounds, see: Dori & Ziolo (1973 ▶); Huynh *et al.* (2003 ▶); Krivopalov & Shkurko (2005 ▶); Meyer *et al.* (1998 ▶); Padwa (1976 ▶).
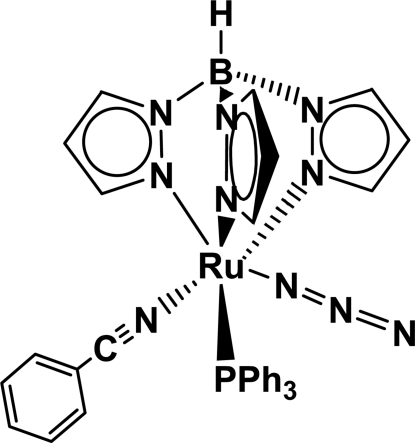


## Experimental

### 

#### Crystal data

[Ru(C_9_H_10_BN_6_)(N_3_)(C_7_H_5_N)(C_18_H_15_P)]
*M* *_r_* = 721.53
Triclinic, 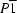

*a* = 11.1888 (2) Å
*b* = 16.2588 (3) Å
*c* = 18.9944 (4) Å
α = 109.588 (1)°
β = 91.930 (1)°
γ = 90.823 (1)°
*V* = 3252.37 (11) Å^3^
*Z* = 4
Mo *K*α radiation
μ = 0.57 mm^−1^
*T* = 200 K
0.20 × 0.08 × 0.03 mm

#### Data collection

Nonius KappaCCD diffractometer
Absorption correction: multi-scan (*DENZO*/*SCALEPACK*; Otwinowski & Minor, 1997 ▶) *T*_min_ = 0.894, *T*_max_ = 0.983
28844 measured reflections
11501 independent reflections
8347 reflections with *I* > 2σ(*I*)
*R*_int_ = 0.049

#### Refinement

*R*[*F* ^2^ > 2σ(*F* ^2^)] = 0.041
*wR*(*F* ^2^) = 0.093
*S* = 1.01
11501 reflections
847 parameters
H-atom parameters constrained
Δρ_max_ = 0.56 e Å^−3^
Δρ_min_ = −0.48 e Å^−3^

### 

Data collection: *COLLECT* (Nonius, 1998 ▶); cell refinement: *DENZO*/*SCALEPACK* (Otwinowski & Minor, 1997 ▶); data reduction: *DENZO*/*SCALEPACK*; program(s) used to solve structure: *SHELXS97* (Sheldrick, 2008 ▶); program(s) used to refine structure: *SHELXL97* (Sheldrick, 2008 ▶); molecular graphics: *ORTEP-3* (Farrugia, 1997 ▶); software used to prepare material for publication: *WinGX* (Farrugia, 1999 ▶).

## Supplementary Material

Crystal structure: contains datablocks I, global. DOI: 10.1107/S1600536810021513/hy2311sup1.cif

Structure factors: contains datablocks I. DOI: 10.1107/S1600536810021513/hy2311Isup2.hkl

Additional supplementary materials:  crystallographic information; 3D view; checkCIF report

## Figures and Tables

**Table 1:** Selected bond lengths (Å)

Ru1—N1	2.122 (3)
Ru1—N3	2.075 (3)
Ru1—N5	2.086 (3)
Ru1—N7	1.984 (3)
Ru1—N8	2.119 (3)
Ru1—P1	2.3068 (11)
Ru2—N11	2.109 (3)
Ru2—N13	2.083 (3)
Ru2—N15	2.077 (3)
Ru2—N17	1.983 (3)
Ru2—N18	2.108 (3)
Ru2—P2	2.3204 (10)

**Fig. 1.**
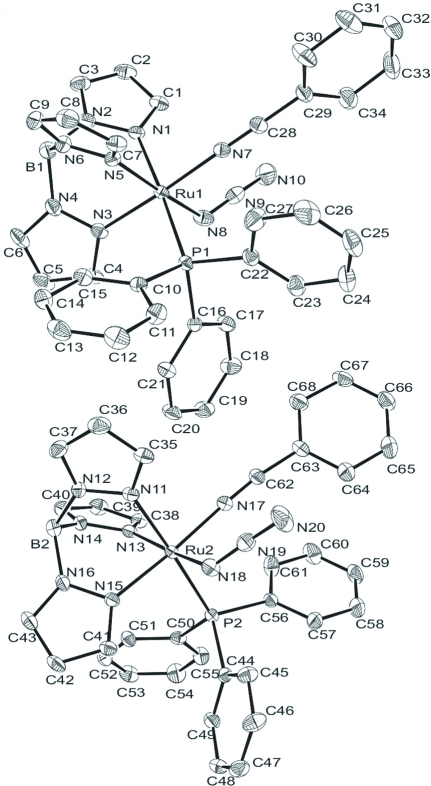
Molecular structure of the title compound. Displacement ellipsoids are drawn at the 30% probability level.

## supplementary crystallographic information

### Comment

Organic azides are synthetically helpful reagents. Amongst many transformations, perhaps the most important one is the 1,3-dipolar cycloaddition reactions with alkynes to synthesize triazoles (Padwa, 1976). Triazoles are nitrogen heteroarenes, which have found a range of significant applications in pharmaceutical and agricultural industries (Krivopalov & Shkurko, 2005). On the other hand, the azide anion, N_3_^-^ , is a versatile ligand because it shows a variety of coordination modes, e.g. end-on monodentate, one-end bridging and end-to-end bridging modes, and also because its complexes exhibit interesting thermal and photochemical reactivities (Dori & Ziolo, 1973; Huynh *et al.*, 2003; Meyer *et al.*, 1998).

The title compound contains two crystallographically distinct molecules (Fig. 1, Table 1). The azide groups are almost linear [N8—N9—N10 = 176.8 (4), N18—N19—N20 = 175.7 (4)°] and are coordinated to Ru with Ru—N—N angles of 123.8 (3) and 125.9 (3)°. There is a small difference between the N—N distances [1.172 (4), 1.188 (4) and 1.173 (4), 1.172 (4) Å]. The Ru1—N7 and N7—C28 bond lengths of 1.983 (3) and 1.150 (4) Å correspond to a single Ru—N bond and a C≡N bond. The N7—C28—C29 of angle 179.0 (4)° indicates an *sp* hybridization as expected.

### Experimental

To a solution of [RuN_3_(htpb)(tpp)_2_] (htpb = hydridotripyrazolylborate, tpp = triphenylphosphine) in 30 ml of THF was added 10 ml of PhCN. The solution was stirred for 22 h, with color changed from yellow to orange-yellow, and then the solution was filtered through Celite. The solvent of the filtrate was removed under vacuum, and the residue was washed with *n*-hexane to give the title compound. The bright-yellow crystals of the title compound for X-ray structure analysis were obtained by slow diffusion of diethyl ether into a CH_2_Cl_2_ solution of the title compound at 0°C for 3 d.

### Refinement

H atoms were placed in idealized positions and constrained to ride on their parent atoms, with C—H = 0.95 and B—H = 1.00 Å and *U*_iso_(H) = 1.2*U*_eq_(C, B).

### Crystal data

**Table:**

[Ru(C_9_H_10_BN_6_)(N_3_)(C_7_H_5_N)(C_18_H_15_P)]	*Z* = 4
*M**_r_* = 721.53	*F*(000) = 1472
Triclinic, *P*1	*D*_x_ = 1.474 Mg m^−^^3^
Hall symbol: -P 1	Mo *K*α radiation, λ = 0.71073 Å
*a* = 11.1888 (2) Å	Cell parameters from 5430 reflections
*b* = 16.2588 (3) Å	θ = 2.7–25.0°
*c* = 18.9944 (4) Å	µ = 0.57 mm^−^^1^
α = 109.588 (1)°	*T* = 200 K
β = 91.930 (1)°	Prism, yellow
γ = 90.823 (1)°	0.20 × 0.08 × 0.03 mm
*V* = 3252.37 (11) Å^3^	

### Data collection

**Table:**

Nonius KappaCCD diffractometer	11501 independent reflections
Radiation source: fine-focus sealed tube	8347 reflections with *I* > 2σ(*I*)
graphite	*R*_int_ = 0.049
Detector resolution: 9 pixels mm^-1^	θ_max_ = 25.0°, θ_min_ = 1.1°
ω and φ scans	*h* = −13→13
Absorption correction: multi-scan (*DENZO*/*SCALEPACK*; Otwinowski & Minor, 1997)	*k* = −14→19
*T*_min_ = 0.894, *T*_max_ = 0.983	*l* = −22→22
28844 measured reflections	

### Refinement

**Table:**

Refinement on *F*^2^	Primary atom site location: structure-invariant direct methods
Least-squares matrix: full	Secondary atom site location: difference Fourier map
*R*[*F*^2^ > 2σ(*F*^2^)] = 0.041	Hydrogen site location: inferred from neighbouring sites
*wR*(*F*^2^) = 0.093	H-atom parameters constrained
*S* = 1.01	*w* = 1/[σ^2^(*F*_o_^2^) + (0.0383*P*)^2^ + 0.0352*P*] where *P* = (*F*_o_^2^ + 2*F*_c_^2^)/3
11501 reflections	(Δ/σ)_max_ = 0.001
847 parameters	Δρ_max_ = 0.56 e Å^−^^3^
0 restraints	Δρ_min_ = −0.48 e Å^−^^3^

### Fractional atomic coordinates and isotropic or equivalent isotropic displacement parameters (Å^2^)

**Table:**

	*x*	*y*	*z*	*U*_iso_*/*U*_eq_	
B1	0.6242 (4)	0.5295 (3)	0.2622 (3)	0.0396 (12)	
H1'	0.5758	0.4750	0.2356	0.048*	
B2	0.8881 (4)	0.5283 (3)	0.7621 (3)	0.0353 (11)	
H2'	0.9355	0.4754	0.7376	0.042*	
C1	0.8553 (3)	0.6230 (3)	0.1793 (2)	0.0350 (10)	
H1	0.9165	0.6654	0.1825	0.042*	
C2	0.8230 (4)	0.5530 (3)	0.1152 (2)	0.0454 (11)	
H2	0.8558	0.5387	0.0672	0.054*	
C3	0.7325 (4)	0.5088 (3)	0.1369 (2)	0.0428 (11)	
H3	0.6909	0.4575	0.1057	0.051*	
C4	0.8330 (4)	0.5572 (3)	0.4207 (2)	0.0406 (11)	
H4	0.8924	0.5893	0.4568	0.049*	
C5	0.7939 (4)	0.4732 (3)	0.4112 (2)	0.0495 (12)	
H5	0.8201	0.4376	0.4391	0.059*	
C6	0.7100 (4)	0.4515 (3)	0.3535 (2)	0.0460 (11)	
H6	0.6672	0.3972	0.3336	0.055*	
C7	0.5004 (3)	0.7446 (3)	0.3399 (2)	0.0356 (10)	
H7	0.5072	0.8060	0.3643	0.043*	
C8	0.3939 (3)	0.6988 (3)	0.3114 (2)	0.0453 (12)	
H8	0.3163	0.7219	0.3124	0.054*	
C9	0.4244 (3)	0.6135 (3)	0.2816 (2)	0.0439 (11)	
H9	0.3709	0.5655	0.2578	0.053*	
C10	0.6223 (3)	0.7708 (2)	0.5168 (2)	0.0276 (9)	
C11	0.5806 (3)	0.8369 (3)	0.5780 (2)	0.0407 (10)	
H11	0.6194	0.8928	0.5943	0.049*	
C12	0.4826 (4)	0.8216 (3)	0.6155 (3)	0.0484 (12)	
H12	0.4551	0.8670	0.6574	0.058*	
C13	0.4256 (3)	0.7412 (3)	0.5921 (3)	0.0473 (12)	
H13	0.3590	0.7308	0.6179	0.057*	
C14	0.4647 (3)	0.6759 (3)	0.5314 (2)	0.0448 (11)	
H14	0.4242	0.6206	0.5149	0.054*	
C15	0.5639 (3)	0.6901 (3)	0.4936 (2)	0.0368 (10)	
H15	0.5911	0.6442	0.4520	0.044*	
C16	0.8749 (3)	0.7677 (2)	0.5389 (2)	0.0255 (8)	
C17	0.9956 (3)	0.7738 (3)	0.5255 (2)	0.0375 (10)	
H17	1.0184	0.7898	0.4840	0.045*	
C18	1.0829 (3)	0.7568 (3)	0.5716 (2)	0.0444 (11)	
H18	1.1650	0.7633	0.5628	0.053*	
C19	1.0514 (3)	0.7305 (3)	0.6305 (2)	0.0423 (11)	
H19	1.1114	0.7182	0.6618	0.051*	
C20	0.9336 (3)	0.7222 (3)	0.6434 (2)	0.0389 (10)	
H20	0.9117	0.7033	0.6835	0.047*	
C21	0.8452 (3)	0.7411 (2)	0.5985 (2)	0.0329 (9)	
H21	0.7635	0.7358	0.6087	0.039*	
C22	0.7704 (3)	0.9056 (2)	0.4965 (2)	0.0312 (9)	
C23	0.8647 (3)	0.9581 (3)	0.5366 (2)	0.0403 (10)	
H23	0.9298	0.9327	0.5545	0.048*	
C24	0.8653 (4)	1.0487 (3)	0.5513 (3)	0.0512 (12)	
H24	0.9314	1.0838	0.5783	0.061*	
C25	0.7734 (4)	1.0862 (3)	0.5275 (3)	0.0564 (13)	
H25	0.7741	1.1477	0.5384	0.068*	
C26	0.6797 (5)	1.0362 (3)	0.4877 (3)	0.0626 (15)	
H26	0.6152	1.0630	0.4706	0.075*	
C27	0.6769 (4)	0.9461 (3)	0.4717 (3)	0.0546 (13)	
H27	0.6107	0.9120	0.4437	0.066*	
C28	0.7948 (3)	0.8687 (3)	0.2999 (2)	0.0340 (9)	
C29	0.8049 (3)	0.9435 (3)	0.2768 (2)	0.0352 (10)	
C30	0.7032 (4)	0.9777 (3)	0.2551 (3)	0.0586 (14)	
H30	0.6270	0.9522	0.2571	0.070*	
C31	0.7110 (5)	1.0477 (3)	0.2310 (3)	0.0637 (15)	
H31	0.6405	1.0703	0.2159	0.076*	
C32	0.8192 (5)	1.0851 (3)	0.2286 (3)	0.0668 (15)	
H32	0.8241	1.1340	0.2119	0.080*	
C33	0.9201 (5)	1.0533 (3)	0.2497 (3)	0.0781 (17)	
H33	0.9955	1.0797	0.2475	0.094*	
C34	0.9145 (4)	0.9819 (3)	0.2750 (3)	0.0575 (13)	
H34	0.9853	0.9602	0.2906	0.069*	
C35	0.6565 (3)	0.6144 (3)	0.6709 (2)	0.0355 (10)	
H35	0.5964	0.6553	0.6717	0.043*	
C36	0.6849 (4)	0.5451 (3)	0.6086 (2)	0.0480 (11)	
H36	0.6500	0.5302	0.5595	0.058*	
C37	0.7734 (4)	0.5024 (3)	0.6324 (2)	0.0459 (11)	
H37	0.8112	0.4513	0.6024	0.055*	
C38	1.0176 (3)	0.7460 (3)	0.8471 (2)	0.0349 (10)	
H38	1.0124	0.8069	0.8727	0.042*	
C39	1.1234 (3)	0.7041 (3)	0.8214 (2)	0.0413 (11)	
H39	1.2015	0.7294	0.8256	0.050*	
C40	1.0891 (3)	0.6182 (3)	0.7887 (2)	0.0397 (11)	
H40	1.1411	0.5719	0.7657	0.048*	
C41	0.6899 (3)	0.5506 (2)	0.9147 (2)	0.0310 (9)	
H41	0.6315	0.5807	0.9483	0.037*	
C42	0.7323 (3)	0.4688 (3)	0.9094 (2)	0.0378 (10)	
H42	0.7111	0.4338	0.9384	0.045*	
C43	0.8113 (3)	0.4499 (3)	0.8530 (2)	0.0370 (10)	
H43	0.8548	0.3977	0.8353	0.044*	
C44	0.6426 (3)	0.7564 (2)	1.0298 (2)	0.0268 (8)	
C45	0.5213 (3)	0.7460 (3)	1.0065 (2)	0.0384 (10)	
H45	0.4959	0.7569	0.9623	0.046*	
C46	0.4381 (3)	0.7198 (3)	1.0481 (2)	0.0469 (12)	
H46	0.3563	0.7118	1.0316	0.056*	
C47	0.4734 (4)	0.7054 (3)	1.1133 (2)	0.0436 (11)	
H47	0.4164	0.6872	1.1414	0.052*	
C48	0.5904 (3)	0.7175 (3)	1.1369 (2)	0.0416 (11)	
H48	0.6148	0.7084	1.1821	0.050*	
C49	0.6745 (3)	0.7428 (3)	1.0960 (2)	0.0352 (10)	
H49	0.7557	0.7510	1.1136	0.042*	
C50	0.8942 (3)	0.7713 (2)	1.0188 (2)	0.0282 (9)	
C51	0.9526 (3)	0.6919 (2)	0.9966 (2)	0.0313 (9)	
H51	0.9222	0.6448	0.9546	0.038*	
C52	1.0545 (3)	0.6817 (3)	1.0357 (2)	0.0394 (10)	
H52	1.0936	0.6274	1.0203	0.047*	
C53	1.1003 (3)	0.7491 (3)	1.0967 (2)	0.0434 (11)	
H53	1.1707	0.7417	1.1231	0.052*	
C54	1.0427 (4)	0.8275 (3)	1.1190 (2)	0.0483 (12)	
H54	1.0734	0.8743	1.1611	0.058*	
C55	0.9406 (3)	0.8385 (3)	1.0806 (2)	0.0381 (10)	
H55	0.9016	0.8928	1.0967	0.046*	
C56	0.7436 (3)	0.8994 (2)	0.9921 (2)	0.0271 (8)	
C57	0.6509 (4)	0.9475 (3)	1.0291 (2)	0.0450 (11)	
H57	0.5875	0.9187	1.0443	0.054*	
C58	0.6477 (4)	1.0375 (3)	1.0450 (3)	0.0584 (14)	
H58	0.5831	1.0693	1.0715	0.070*	
C59	0.7375 (4)	1.0806 (3)	1.0225 (2)	0.0468 (11)	
H59	0.7353	1.1419	1.0329	0.056*	
C60	0.8305 (4)	1.0334 (3)	0.9845 (3)	0.0518 (13)	
H60	0.8935	1.0621	0.9688	0.062*	
C61	0.8321 (3)	0.9440 (3)	0.9694 (3)	0.0441 (11)	
H61	0.8962	0.9121	0.9425	0.053*	
C62	0.7215 (3)	0.8628 (3)	0.7954 (2)	0.0313 (9)	
C63	0.6984 (3)	0.9433 (3)	0.7822 (2)	0.0338 (9)	
C64	0.6199 (4)	0.9992 (3)	0.8289 (3)	0.0504 (12)	
H64	0.5836	0.9836	0.8673	0.060*	
C65	0.5950 (4)	1.0775 (3)	0.8194 (3)	0.0603 (14)	
H65	0.5392	1.1152	0.8502	0.072*	
C66	0.6503 (4)	1.1015 (3)	0.7655 (3)	0.0512 (12)	
H66	0.6338	1.1562	0.7598	0.061*	
C67	0.7275 (4)	1.0481 (3)	0.7207 (2)	0.0464 (11)	
H67	0.7644	1.0650	0.6832	0.056*	
C68	0.7543 (4)	0.9678 (3)	0.7286 (2)	0.0434 (11)	
H68	0.8102	0.9307	0.6974	0.052*	
N1	0.7875 (3)	0.6218 (2)	0.23571 (17)	0.0298 (7)	
N2	0.7132 (3)	0.5507 (2)	0.20954 (18)	0.0345 (8)	
N3	0.7748 (3)	0.5870 (2)	0.37157 (17)	0.0307 (8)	
N4	0.6987 (3)	0.5207 (2)	0.32976 (18)	0.0361 (8)	
N5	0.5921 (2)	0.6903 (2)	0.32828 (17)	0.0295 (7)	
N6	0.5438 (3)	0.6089 (2)	0.29169 (18)	0.0356 (8)	
N7	0.7850 (2)	0.8086 (2)	0.31847 (17)	0.0285 (7)	
N8	0.9664 (3)	0.7047 (2)	0.35704 (19)	0.0387 (9)	
N9	1.0281 (3)	0.7336 (2)	0.3220 (2)	0.0391 (8)	
N10	1.0951 (3)	0.7611 (3)	0.2875 (2)	0.0607 (11)	
N11	0.7258 (2)	0.6156 (2)	0.73002 (17)	0.0291 (7)	
N12	0.7979 (3)	0.5453 (2)	0.70548 (18)	0.0324 (8)	
N13	0.9242 (2)	0.6894 (2)	0.83116 (17)	0.0279 (7)	
N14	0.9701 (2)	0.6093 (2)	0.79404 (17)	0.0300 (7)	
N15	0.7429 (2)	0.58089 (19)	0.86601 (16)	0.0270 (7)	
N16	0.8175 (2)	0.5169 (2)	0.82667 (18)	0.0307 (7)	
N17	0.7334 (2)	0.8024 (2)	0.81263 (17)	0.0288 (7)	
N18	0.5513 (3)	0.6914 (2)	0.83975 (18)	0.0355 (8)	
N19	0.4879 (3)	0.7312 (2)	0.81356 (19)	0.0370 (8)	
N20	0.4184 (3)	0.7681 (3)	0.7884 (3)	0.0688 (13)	
P1	0.76176 (8)	0.78578 (6)	0.47329 (5)	0.0251 (2)	
P2	0.75346 (8)	0.78065 (6)	0.96896 (5)	0.0242 (2)	
Ru1	0.77697 (2)	0.703233 (19)	0.348879 (17)	0.02482 (9)	
Ru2	0.73983 (2)	0.697064 (19)	0.842995 (16)	0.02291 (9)	

### Atomic displacement parameters (Å^2^)

**Table:**

	*U*^11^	*U*^22^	*U*^33^	*U*^12^	*U*^13^	*U*^23^
B1	0.049 (3)	0.036 (3)	0.032 (3)	−0.006 (2)	0.001 (2)	0.011 (2)
B2	0.039 (2)	0.030 (3)	0.038 (3)	0.007 (2)	0.003 (2)	0.013 (2)
C1	0.037 (2)	0.043 (3)	0.030 (2)	0.0103 (19)	0.0069 (18)	0.017 (2)
C2	0.055 (3)	0.056 (3)	0.026 (3)	0.016 (2)	0.009 (2)	0.014 (2)
C3	0.060 (3)	0.035 (3)	0.029 (3)	0.009 (2)	−0.006 (2)	0.006 (2)
C4	0.058 (3)	0.038 (3)	0.031 (3)	0.022 (2)	0.007 (2)	0.017 (2)
C5	0.086 (3)	0.029 (3)	0.041 (3)	0.016 (2)	0.010 (3)	0.020 (2)
C6	0.071 (3)	0.031 (3)	0.046 (3)	0.008 (2)	0.018 (2)	0.022 (2)
C7	0.037 (2)	0.040 (3)	0.032 (2)	0.0067 (19)	−0.0003 (18)	0.015 (2)
C8	0.029 (2)	0.068 (3)	0.046 (3)	0.004 (2)	0.0015 (19)	0.029 (3)
C9	0.034 (2)	0.057 (3)	0.044 (3)	−0.018 (2)	−0.0098 (19)	0.023 (2)
C10	0.0270 (19)	0.032 (2)	0.025 (2)	0.0027 (17)	−0.0008 (16)	0.0111 (19)
C11	0.038 (2)	0.040 (3)	0.040 (3)	0.0025 (19)	0.0050 (19)	0.008 (2)
C12	0.044 (2)	0.058 (3)	0.045 (3)	0.015 (2)	0.020 (2)	0.018 (3)
C13	0.030 (2)	0.073 (4)	0.052 (3)	0.004 (2)	0.009 (2)	0.039 (3)
C14	0.041 (2)	0.054 (3)	0.043 (3)	−0.012 (2)	0.002 (2)	0.022 (3)
C15	0.040 (2)	0.041 (3)	0.031 (2)	−0.0008 (19)	0.0042 (18)	0.014 (2)
C16	0.0306 (19)	0.021 (2)	0.024 (2)	0.0014 (16)	−0.0003 (16)	0.0071 (17)
C17	0.035 (2)	0.048 (3)	0.036 (3)	0.0012 (19)	0.0014 (18)	0.022 (2)
C18	0.030 (2)	0.064 (3)	0.042 (3)	0.004 (2)	−0.0005 (19)	0.022 (3)
C19	0.044 (2)	0.046 (3)	0.040 (3)	0.010 (2)	−0.011 (2)	0.020 (2)
C20	0.044 (2)	0.046 (3)	0.037 (3)	0.004 (2)	−0.0021 (19)	0.028 (2)
C21	0.037 (2)	0.035 (2)	0.030 (2)	0.0058 (18)	0.0060 (17)	0.015 (2)
C22	0.035 (2)	0.029 (2)	0.030 (2)	0.0019 (18)	0.0069 (17)	0.0103 (19)
C23	0.039 (2)	0.039 (3)	0.043 (3)	−0.001 (2)	0.008 (2)	0.014 (2)
C24	0.055 (3)	0.033 (3)	0.065 (4)	−0.004 (2)	0.016 (2)	0.015 (3)
C25	0.075 (3)	0.032 (3)	0.068 (4)	0.007 (3)	0.025 (3)	0.022 (3)
C26	0.080 (4)	0.044 (3)	0.070 (4)	0.028 (3)	0.002 (3)	0.027 (3)
C27	0.063 (3)	0.032 (3)	0.063 (3)	0.008 (2)	−0.018 (2)	0.011 (2)
C28	0.035 (2)	0.035 (3)	0.034 (3)	0.0047 (18)	0.0022 (18)	0.014 (2)
C29	0.047 (2)	0.028 (2)	0.033 (2)	0.0030 (19)	0.0057 (19)	0.013 (2)
C30	0.053 (3)	0.066 (4)	0.079 (4)	0.015 (3)	0.014 (3)	0.053 (3)
C31	0.081 (4)	0.059 (4)	0.067 (4)	0.028 (3)	0.018 (3)	0.040 (3)
C32	0.097 (4)	0.042 (3)	0.073 (4)	−0.003 (3)	−0.005 (3)	0.035 (3)
C33	0.077 (4)	0.058 (4)	0.112 (5)	−0.030 (3)	−0.017 (3)	0.048 (4)
C34	0.054 (3)	0.051 (3)	0.077 (4)	−0.006 (2)	−0.014 (3)	0.036 (3)
C35	0.039 (2)	0.039 (3)	0.032 (2)	−0.0059 (19)	−0.0058 (18)	0.018 (2)
C36	0.067 (3)	0.046 (3)	0.028 (3)	−0.010 (2)	−0.008 (2)	0.010 (2)
C37	0.070 (3)	0.032 (3)	0.029 (3)	0.001 (2)	0.011 (2)	0.002 (2)
C38	0.039 (2)	0.036 (3)	0.034 (3)	−0.0002 (19)	0.0048 (18)	0.016 (2)
C39	0.025 (2)	0.058 (3)	0.048 (3)	0.000 (2)	0.0054 (19)	0.027 (3)
C40	0.030 (2)	0.054 (3)	0.045 (3)	0.013 (2)	0.0120 (19)	0.028 (2)
C41	0.036 (2)	0.030 (2)	0.028 (2)	−0.0052 (17)	0.0003 (17)	0.0122 (19)
C42	0.051 (2)	0.028 (2)	0.041 (3)	−0.0063 (19)	−0.004 (2)	0.022 (2)
C43	0.045 (2)	0.027 (2)	0.043 (3)	0.0004 (18)	−0.006 (2)	0.018 (2)
C44	0.0307 (19)	0.020 (2)	0.028 (2)	−0.0006 (16)	0.0045 (16)	0.0065 (18)
C45	0.032 (2)	0.051 (3)	0.033 (2)	−0.0027 (19)	0.0017 (18)	0.015 (2)
C46	0.033 (2)	0.064 (3)	0.041 (3)	−0.008 (2)	0.0066 (19)	0.013 (3)
C47	0.047 (3)	0.040 (3)	0.046 (3)	−0.005 (2)	0.018 (2)	0.016 (2)
C48	0.050 (3)	0.045 (3)	0.040 (3)	0.007 (2)	0.010 (2)	0.027 (2)
C49	0.034 (2)	0.037 (3)	0.040 (3)	0.0056 (18)	0.0035 (18)	0.020 (2)
C50	0.0230 (18)	0.037 (2)	0.028 (2)	−0.0030 (17)	−0.0002 (16)	0.016 (2)
C51	0.033 (2)	0.030 (2)	0.032 (2)	0.0012 (17)	−0.0052 (17)	0.0125 (19)
C52	0.035 (2)	0.043 (3)	0.044 (3)	0.012 (2)	−0.0019 (19)	0.019 (2)
C53	0.030 (2)	0.061 (3)	0.046 (3)	−0.001 (2)	−0.0085 (19)	0.028 (3)
C54	0.047 (3)	0.051 (3)	0.043 (3)	−0.003 (2)	−0.016 (2)	0.011 (2)
C55	0.037 (2)	0.034 (3)	0.039 (3)	0.0001 (19)	−0.0072 (19)	0.008 (2)
C56	0.034 (2)	0.023 (2)	0.023 (2)	−0.0001 (16)	−0.0029 (16)	0.0076 (18)
C57	0.055 (3)	0.034 (3)	0.052 (3)	0.010 (2)	0.022 (2)	0.021 (2)
C58	0.079 (3)	0.035 (3)	0.068 (4)	0.019 (3)	0.027 (3)	0.024 (3)
C59	0.067 (3)	0.023 (2)	0.049 (3)	0.002 (2)	−0.004 (2)	0.011 (2)
C60	0.046 (3)	0.038 (3)	0.080 (4)	−0.009 (2)	0.002 (2)	0.030 (3)
C61	0.033 (2)	0.029 (3)	0.069 (3)	−0.0016 (18)	0.004 (2)	0.015 (2)
C62	0.032 (2)	0.032 (2)	0.031 (2)	0.0018 (17)	−0.0019 (17)	0.013 (2)
C63	0.037 (2)	0.033 (2)	0.036 (3)	−0.0023 (18)	−0.0058 (18)	0.017 (2)
C64	0.062 (3)	0.042 (3)	0.055 (3)	0.013 (2)	0.014 (2)	0.025 (3)
C65	0.074 (3)	0.041 (3)	0.071 (4)	0.020 (3)	0.014 (3)	0.023 (3)
C66	0.073 (3)	0.036 (3)	0.051 (3)	0.009 (2)	−0.001 (2)	0.022 (3)
C67	0.066 (3)	0.040 (3)	0.041 (3)	−0.007 (2)	−0.001 (2)	0.025 (2)
C68	0.052 (3)	0.040 (3)	0.041 (3)	0.000 (2)	0.001 (2)	0.019 (2)
N1	0.0373 (17)	0.030 (2)	0.0233 (19)	0.0038 (15)	−0.0015 (14)	0.0109 (16)
N2	0.0454 (19)	0.030 (2)	0.029 (2)	0.0013 (16)	−0.0012 (15)	0.0106 (17)
N3	0.0379 (18)	0.028 (2)	0.0283 (19)	0.0074 (15)	0.0050 (14)	0.0109 (16)
N4	0.048 (2)	0.028 (2)	0.035 (2)	0.0013 (16)	0.0074 (16)	0.0126 (17)
N5	0.0274 (16)	0.035 (2)	0.0277 (19)	−0.0018 (14)	−0.0032 (13)	0.0125 (16)
N6	0.0351 (18)	0.039 (2)	0.033 (2)	−0.0065 (16)	−0.0018 (15)	0.0128 (18)
N7	0.0258 (16)	0.032 (2)	0.0283 (19)	0.0035 (14)	−0.0006 (13)	0.0117 (17)
N8	0.0350 (18)	0.052 (2)	0.033 (2)	0.0108 (17)	0.0060 (15)	0.0187 (19)
N9	0.0322 (18)	0.041 (2)	0.035 (2)	0.0069 (16)	−0.0022 (16)	0.0016 (19)
N10	0.043 (2)	0.078 (3)	0.062 (3)	−0.011 (2)	0.006 (2)	0.026 (3)
N11	0.0330 (17)	0.032 (2)	0.0250 (19)	−0.0014 (14)	−0.0004 (14)	0.0126 (16)
N12	0.0441 (19)	0.0240 (19)	0.029 (2)	0.0041 (15)	0.0069 (15)	0.0081 (16)
N13	0.0297 (16)	0.032 (2)	0.0269 (19)	0.0013 (14)	0.0017 (13)	0.0162 (16)
N14	0.0329 (17)	0.0288 (19)	0.0320 (19)	0.0071 (14)	0.0062 (14)	0.0142 (16)
N15	0.0336 (16)	0.0242 (18)	0.0240 (18)	−0.0008 (14)	−0.0011 (13)	0.0094 (15)
N16	0.0345 (17)	0.0252 (19)	0.035 (2)	0.0040 (14)	0.0007 (14)	0.0132 (16)
N17	0.0294 (16)	0.031 (2)	0.0283 (19)	0.0000 (14)	0.0002 (14)	0.0123 (16)
N18	0.0305 (17)	0.040 (2)	0.041 (2)	0.0000 (15)	0.0005 (15)	0.0198 (18)
N19	0.0298 (17)	0.042 (2)	0.039 (2)	−0.0004 (16)	0.0060 (15)	0.0128 (19)
N20	0.040 (2)	0.087 (3)	0.101 (4)	0.022 (2)	0.004 (2)	0.059 (3)
P1	0.0258 (5)	0.0255 (6)	0.0269 (6)	0.0018 (4)	0.0011 (4)	0.0124 (5)
P2	0.0241 (5)	0.0239 (6)	0.0259 (6)	0.0009 (4)	0.0003 (4)	0.0103 (5)
Ru1	0.02614 (16)	0.02651 (19)	0.02497 (19)	0.00370 (13)	0.00169 (12)	0.01262 (15)
Ru2	0.02437 (15)	0.02269 (18)	0.02441 (19)	0.00007 (12)	0.00033 (12)	0.01164 (15)

### Geometric parameters (Å, °)

**Table:**

B1—N6	1.539 (5)	C38—N13	1.339 (5)
B1—N2	1.548 (5)	C38—C39	1.394 (5)
B1—N4	1.555 (5)	C38—H38	0.9500
B1—H1'	1.0000	C39—C40	1.368 (6)
B2—N14	1.528 (5)	C39—H39	0.9500
B2—N16	1.542 (5)	C40—N14	1.349 (4)
B2—N12	1.545 (5)	C40—H40	0.9500
B2—H2'	1.0000	C41—N15	1.336 (4)
C1—N1	1.339 (4)	C41—C42	1.390 (5)
C1—C2	1.392 (6)	C41—H41	0.9500
C1—H1	0.9500	C42—C43	1.368 (5)
C2—C3	1.386 (6)	C42—H42	0.9500
C2—H2	0.9500	C43—N16	1.344 (4)
C3—N2	1.345 (5)	C43—H43	0.9500
C3—H3	0.9500	C44—C49	1.384 (5)
C4—N3	1.341 (4)	C44—C45	1.404 (5)
C4—C5	1.379 (6)	C44—P2	1.849 (3)
C4—H4	0.9500	C45—C46	1.391 (5)
C5—C6	1.366 (6)	C45—H45	0.9500
C5—H5	0.9500	C46—C47	1.382 (6)
C6—N4	1.352 (4)	C46—H46	0.9500
C6—H6	0.9500	C47—C48	1.360 (6)
C7—N5	1.338 (4)	C47—H47	0.9500
C7—C8	1.389 (5)	C48—C49	1.382 (5)
C7—H7	0.9500	C48—H48	0.9500
C8—C9	1.364 (6)	C49—H49	0.9500
C8—H8	0.9500	C50—C55	1.387 (5)
C9—N6	1.350 (4)	C50—C51	1.396 (5)
C9—H9	0.9500	C50—P2	1.845 (3)
C10—C15	1.383 (5)	C51—C52	1.382 (5)
C10—C11	1.391 (5)	C51—H51	0.9500
C10—P1	1.840 (3)	C52—C53	1.378 (6)
C11—C12	1.391 (5)	C52—H52	0.9500
C11—H11	0.9500	C53—C54	1.378 (5)
C12—C13	1.372 (6)	C53—H53	0.9500
C12—H12	0.9500	C54—C55	1.381 (5)
C13—C14	1.368 (6)	C54—H54	0.9500
C13—H13	0.9500	C55—H55	0.9500
C14—C15	1.399 (5)	C56—C57	1.373 (5)
C14—H14	0.9500	C56—C61	1.381 (5)
C15—H15	0.9500	C56—P2	1.837 (4)
C16—C17	1.392 (5)	C57—C58	1.392 (5)
C16—C21	1.389 (5)	C57—H57	0.9500
C16—P1	1.842 (3)	C58—C59	1.376 (6)
C17—C18	1.383 (5)	C58—H58	0.9500
C17—H17	0.9500	C59—C60	1.376 (5)
C18—C19	1.378 (5)	C59—H59	0.9500
C18—H18	0.9500	C60—C61	1.385 (5)
C19—C20	1.361 (5)	C60—H60	0.9500
C19—H19	0.9500	C61—H61	0.9500
C20—C21	1.387 (5)	C62—N17	1.143 (4)
C20—H20	0.9500	C62—C63	1.438 (5)
C21—H21	0.9500	C63—C68	1.376 (5)
C22—C23	1.380 (5)	C63—C64	1.385 (5)
C22—C27	1.396 (5)	C64—C65	1.376 (5)
C22—P1	1.848 (4)	C64—H64	0.9500
C23—C24	1.404 (5)	C65—C66	1.374 (6)
C23—H23	0.9500	C65—H65	0.9500
C24—C25	1.345 (6)	C66—C67	1.341 (6)
C24—H24	0.9500	C66—H66	0.9500
C25—C26	1.358 (7)	C67—C68	1.398 (5)
C25—H25	0.9500	C67—H67	0.9500
C26—C27	1.392 (6)	C68—H68	0.9500
C26—H26	0.9500	N1—N2	1.355 (4)
C27—H27	0.9500	N3—N4	1.368 (4)
C28—N7	1.150 (4)	N5—N6	1.366 (4)
C28—C29	1.429 (5)	N8—N9	1.172 (4)
C29—C34	1.374 (5)	N9—N10	1.188 (4)
C29—C30	1.384 (5)	N11—N12	1.367 (4)
C30—C31	1.364 (6)	N13—N14	1.373 (4)
C30—H30	0.9500	N15—N16	1.372 (4)
C31—C32	1.356 (7)	N18—N19	1.173 (4)
C31—H31	0.9500	N19—N20	1.172 (4)
C32—C33	1.353 (7)	Ru1—N1	2.122 (3)
C32—H32	0.9500	Ru1—N3	2.075 (3)
C33—C34	1.398 (6)	Ru1—N5	2.086 (3)
C33—H33	0.9500	Ru1—N7	1.984 (3)
C34—H34	0.9500	Ru1—N8	2.119 (3)
C35—N11	1.337 (4)	Ru1—P1	2.3068 (11)
C35—C36	1.383 (5)	Ru2—N11	2.109 (3)
C35—H35	0.9500	Ru2—N13	2.083 (3)
C36—C37	1.366 (6)	Ru2—N15	2.077 (3)
C36—H36	0.9500	Ru2—N17	1.983 (3)
C37—N12	1.345 (5)	Ru2—N18	2.108 (3)
C37—H37	0.9500	Ru2—P2	2.3204 (10)
			
N6—B1—N2	107.2 (3)	C48—C47—C46	119.4 (4)
N6—B1—N4	107.9 (3)	C48—C47—H47	120.3
N2—B1—N4	107.3 (3)	C46—C47—H47	120.3
N6—B1—H1'	111.4	C47—C48—C49	120.8 (4)
N2—B1—H1'	111.4	C47—C48—H48	119.6
N4—B1—H1'	111.4	C49—C48—H48	119.6
N14—B2—N16	108.1 (3)	C48—C49—C44	121.3 (4)
N14—B2—N12	107.9 (3)	C48—C49—H49	119.3
N16—B2—N12	108.1 (3)	C44—C49—H49	119.3
N14—B2—H2'	110.9	C55—C50—C51	118.4 (3)
N16—B2—H2'	110.9	C55—C50—P2	122.5 (3)
N12—B2—H2'	110.9	C51—C50—P2	119.0 (3)
N1—C1—C2	110.0 (4)	C52—C51—C50	120.1 (4)
N1—C1—H1	125.0	C52—C51—H51	120.0
C2—C1—H1	125.0	C50—C51—H51	120.0
C3—C2—C1	104.7 (4)	C51—C52—C53	121.0 (4)
C3—C2—H2	127.7	C51—C52—H52	119.5
C1—C2—H2	127.7	C53—C52—H52	119.5
N2—C3—C2	108.6 (4)	C54—C53—C52	119.2 (4)
N2—C3—H3	125.7	C54—C53—H53	120.4
C2—C3—H3	125.7	C52—C53—H53	120.4
N3—C4—C5	110.0 (4)	C53—C54—C55	120.4 (4)
N3—C4—H4	125.0	C53—C54—H54	119.8
C5—C4—H4	125.0	C55—C54—H54	119.8
C6—C5—C4	106.2 (4)	C54—C55—C50	120.9 (4)
C6—C5—H5	126.9	C54—C55—H55	119.5
C4—C5—H5	126.9	C50—C55—H55	119.5
N4—C6—C5	108.0 (4)	C57—C56—C61	117.0 (3)
N4—C6—H6	126.0	C57—C56—P2	124.0 (3)
C5—C6—H6	126.0	C61—C56—P2	119.0 (3)
N5—C7—C8	110.7 (4)	C56—C57—C58	121.6 (4)
N5—C7—H7	124.7	C56—C57—H57	119.2
C8—C7—H7	124.7	C58—C57—H57	119.2
C9—C8—C7	105.4 (4)	C59—C58—C57	120.3 (4)
C9—C8—H8	127.3	C59—C58—H58	119.8
C7—C8—H8	127.3	C57—C58—H58	119.8
N6—C9—C8	108.2 (4)	C58—C59—C60	118.9 (4)
N6—C9—H9	125.9	C58—C59—H59	120.6
C8—C9—H9	125.9	C60—C59—H59	120.6
C15—C10—C11	118.7 (3)	C59—C60—C61	119.8 (4)
C15—C10—P1	119.9 (3)	C59—C60—H60	120.1
C11—C10—P1	121.1 (3)	C61—C60—H60	120.1
C12—C11—C10	120.6 (4)	C56—C61—C60	122.3 (4)
C12—C11—H11	119.7	C56—C61—H61	118.9
C10—C11—H11	119.7	C60—C61—H61	118.9
C13—C12—C11	120.1 (4)	N17—C62—C63	172.8 (4)
C13—C12—H12	119.9	C68—C63—C64	119.9 (4)
C11—C12—H12	119.9	C68—C63—C62	123.1 (4)
C14—C13—C12	120.0 (4)	C64—C63—C62	116.9 (3)
C14—C13—H13	120.0	C65—C64—C63	119.6 (4)
C12—C13—H13	120.0	C65—C64—H64	120.2
C13—C14—C15	120.5 (4)	C63—C64—H64	120.2
C13—C14—H14	119.8	C64—C65—C66	120.3 (4)
C15—C14—H14	119.8	C64—C65—H65	119.8
C10—C15—C14	120.1 (4)	C66—C65—H65	119.8
C10—C15—H15	119.9	C67—C66—C65	120.3 (4)
C14—C15—H15	119.9	C67—C66—H66	119.9
C17—C16—C21	117.8 (3)	C65—C66—H66	119.9
C17—C16—P1	119.3 (3)	C66—C67—C68	120.8 (4)
C21—C16—P1	122.8 (3)	C66—C67—H67	119.6
C18—C17—C16	120.9 (4)	C68—C67—H67	119.6
C18—C17—H17	119.6	C63—C68—C67	119.0 (4)
C16—C17—H17	119.6	C63—C68—H68	120.5
C19—C18—C17	120.3 (4)	C67—C68—H68	120.5
C19—C18—H18	119.8	C1—N1—N2	107.3 (3)
C17—C18—H18	119.8	C1—N1—Ru1	134.2 (3)
C20—C19—C18	119.5 (3)	N2—N1—Ru1	118.6 (2)
C20—C19—H19	120.2	C3—N2—N1	109.4 (3)
C18—C19—H19	120.2	C3—N2—B1	130.5 (4)
C19—C20—C21	120.7 (4)	N1—N2—B1	120.1 (3)
C19—C20—H20	119.7	C4—N3—N4	106.4 (3)
C21—C20—H20	119.7	C4—N3—Ru1	135.1 (3)
C20—C21—C16	120.8 (3)	N4—N3—Ru1	118.5 (2)
C20—C21—H21	119.6	C6—N4—N3	109.4 (3)
C16—C21—H21	119.6	C6—N4—B1	129.8 (4)
C23—C22—C27	117.5 (4)	N3—N4—B1	120.6 (3)
C23—C22—P1	124.0 (3)	C7—N5—N6	105.8 (3)
C27—C22—P1	118.5 (3)	C7—N5—Ru1	136.0 (3)
C22—C23—C24	120.6 (4)	N6—N5—Ru1	118.2 (2)
C22—C23—H23	119.7	C9—N6—N5	109.9 (3)
C24—C23—H23	119.7	C9—N6—B1	128.8 (4)
C25—C24—C23	120.7 (5)	N5—N6—B1	121.0 (3)
C25—C24—H24	119.6	C28—N7—Ru1	176.9 (3)
C23—C24—H24	119.6	N9—N8—Ru1	123.8 (3)
C24—C25—C26	119.9 (5)	N8—N9—N10	176.8 (4)
C24—C25—H25	120.1	C35—N11—N12	106.1 (3)
C26—C25—H25	120.1	C35—N11—Ru2	134.2 (3)
C25—C26—C27	120.7 (4)	N12—N11—Ru2	119.7 (2)
C25—C26—H26	119.6	C37—N12—N11	109.7 (3)
C27—C26—H26	119.6	C37—N12—B2	131.9 (3)
C26—C27—C22	120.5 (5)	N11—N12—B2	118.4 (3)
C26—C27—H27	119.8	C38—N13—N14	105.5 (3)
C22—C27—H27	119.8	C38—N13—Ru2	136.3 (3)
N7—C28—C29	179.0 (4)	N14—N13—Ru2	118.0 (2)
C34—C29—C30	119.1 (4)	C40—N14—N13	109.4 (3)
C34—C29—C28	121.1 (4)	C40—N14—B2	129.6 (3)
C30—C29—C28	119.7 (4)	N13—N14—B2	120.9 (3)
C31—C30—C29	120.8 (4)	C41—N15—N16	106.4 (3)
C31—C30—H30	119.6	C41—N15—Ru2	135.1 (3)
C29—C30—H30	119.6	N16—N15—Ru2	118.5 (2)
C32—C31—C30	120.1 (5)	C43—N16—N15	109.0 (3)
C32—C31—H31	119.9	C43—N16—B2	130.8 (3)
C30—C31—H31	119.9	N15—N16—B2	120.2 (3)
C33—C32—C31	120.4 (5)	C62—N17—Ru2	175.3 (3)
C33—C32—H32	119.8	N19—N18—Ru2	125.9 (3)
C31—C32—H32	119.8	N20—N19—N18	175.7 (4)
C32—C33—C34	120.6 (5)	C10—P1—C16	101.27 (15)
C32—C33—H33	119.7	C10—P1—C22	101.87 (16)
C34—C33—H33	119.7	C16—P1—C22	102.37 (17)
C29—C34—C33	119.0 (4)	C10—P1—Ru1	116.14 (13)
C29—C34—H34	120.5	C16—P1—Ru1	116.39 (11)
C33—C34—H34	120.5	C22—P1—Ru1	116.42 (12)
N11—C35—C36	110.3 (4)	C56—P2—C50	102.06 (17)
N11—C35—H35	124.9	C56—P2—C44	102.77 (16)
C36—C35—H35	124.9	C50—P2—C44	100.64 (16)
C37—C36—C35	105.7 (4)	C56—P2—Ru2	116.66 (12)
C37—C36—H36	127.2	C50—P2—Ru2	115.27 (12)
C35—C36—H36	127.2	C44—P2—Ru2	117.02 (13)
N12—C37—C36	108.2 (4)	N7—Ru1—N3	174.90 (12)
N12—C37—H37	125.9	N7—Ru1—N5	91.69 (11)
C36—C37—H37	125.9	N3—Ru1—N5	89.56 (12)
N13—C38—C39	111.4 (4)	N7—Ru1—N8	89.40 (12)
N13—C38—H38	124.3	N3—Ru1—N8	88.78 (12)
C39—C38—H38	124.3	N5—Ru1—N8	173.12 (13)
C40—C39—C38	104.2 (3)	N7—Ru1—N1	90.41 (12)
C40—C39—H39	127.9	N3—Ru1—N1	84.76 (12)
C38—C39—H39	127.9	N5—Ru1—N1	85.30 (12)
N14—C40—C39	109.4 (3)	N8—Ru1—N1	87.90 (12)
N14—C40—H40	125.3	N7—Ru1—P1	92.34 (9)
C39—C40—H40	125.3	N3—Ru1—P1	92.52 (9)
N15—C41—C42	110.5 (3)	N5—Ru1—P1	93.70 (9)
N15—C41—H41	124.8	N8—Ru1—P1	93.05 (10)
C42—C41—H41	124.8	N1—Ru1—P1	177.11 (9)
C43—C42—C41	104.9 (3)	N17—Ru2—N15	175.44 (12)
C43—C42—H42	127.5	N17—Ru2—N13	91.90 (11)
C41—C42—H42	127.5	N15—Ru2—N13	88.56 (11)
N16—C43—C42	109.2 (3)	N17—Ru2—N18	89.20 (12)
N16—C43—H43	125.4	N15—Ru2—N18	89.72 (11)
C42—C43—H43	125.4	N13—Ru2—N18	171.90 (13)
C49—C44—C45	117.8 (3)	N17—Ru2—N11	90.68 (12)
C49—C44—P2	122.8 (3)	N15—Ru2—N11	84.83 (11)
C45—C44—P2	119.3 (3)	N13—Ru2—N11	85.80 (11)
C46—C45—C44	120.1 (4)	N18—Ru2—N11	86.16 (12)
C46—C45—H45	119.9	N17—Ru2—P2	92.09 (9)
C44—C45—H45	119.9	N15—Ru2—P2	92.39 (9)
C47—C46—C45	120.5 (4)	N13—Ru2—P2	94.69 (9)
C47—C46—H46	119.7	N18—Ru2—P2	93.29 (9)
C45—C46—H46	119.7	N11—Ru2—P2	177.17 (8)
